# Noninvasive ventilation in status asthmaticus in children: levels of evidence

**DOI:** 10.5935/0103-507X.20150065

**Published:** 2015

**Authors:** Paula de Souza Silva, Sérgio Saldanha Menna Barreto

**Affiliations:** 1 Hospital da Criança Conceição, Grupo Hospitalar Conceição - Porto Alegre (RS), Brazil.; 2 Postgraduate Program in Child and Adolescent Health, Faculdade de Medicina, Universidade Federal do Rio Grande do Sul - Porto Alegre (RS), Brazil.; 3 Department of Internal Medicine, Faculdade de Medicina, Universidade Federal do Rio Grande do Sul - Porto Alegre (RS), Brazil.

**Keywords:** Noninvasive ventilation, Bronchial spasmus, Asthma, Status asmathicus, Respiratory insufficiency, Hypercapnia, Child

## Abstract

**Objective:**

To evaluate the quality of available evidence to establish guidelines for the use of noninvasive ventilation for the management of status asthmaticus in children unresponsive to standard treatment.

**Methods:**

Search, selection and analysis of all original articles on asthma and noninvasive ventilation in children, published until September 1, 2014 in all languages in the electronic databases PubMed, Web of Science, Cochrane Library, Scopus and SciELO, located using the search terms: "asthma", "status asthmaticus", "noninvasive ventilation", "Bronchospasm", "continuous positive airway pressure", "child", "infant", "pediatrics", "hypercapnia", "respiratory failure" and the keywords "BIPAP", "CPAP", "Bilevel", "acute asthma" and "near fatal asthma". The articles were assessed based on the levels of evidence of the GRADE system.

**Results:**

Only nine original articles were located; two (22%) articles had level of evidence A, one (11%) had level of evidence B and six (67%) had level of evidence C.

**Conclusion:**

The results suggest that noninvasive ventilation is applicable for the treatment of status asthmaticus in most pediatric patients unresponsive to standard treatment. However, the available evidence cannot be considered as conclusive, as further high-quality research is likely to have an impact on and change the estimate of the effect.

## Abstract (pt)

**Objetivo:**

Avaliar a qualidade das evidências existentes para embasar diretrizes do emprego da ventilação mecânica não invasiva no manejo da crise de asma aguda grave em crianças não responsivas ao tratamento padrão.

**Métodos:**

Busca, seleção e análise de todos os artigos originais sobre asma e ventilação mecânica não invasiva em crianças, publicados até 1º de setembro de 2014, em todos os idiomas, nas bases de dados eletrônicas PubMed, *Web of Science, Cochrane Library*, Scopus e SciELO, encontrados por meio de busca pelos descritores "*asthma*", "*status asthmaticus*", "*noninvasive ventilation*", "*bronchospasm*", "*continuous positive airway pressure*", "*child*", "*infant*", "*pediatrics*", "*hypercapnia*", "*respiratory failure*", e das palavras-chave "BIPAP", "CPAP", "*bilevel*", "*acute asthma*" e "*near fatal asthma*". Os artigos foram qualificados segundo os graus de evidências do Sistema GRADE.

**Resultados:**

Foram obtidos apenas nove artigos originais. Destes, dois (22%) apresentaram nível de evidência A, um (11%) apresentou nível de evidência B e seis (67%) apresentaram nível de evidência C.

**Conclusão:**

Sugere-se que o emprego da ventilação mecânica não invasiva na crise de asma aguda grave em crianças não responsivas ao tratamento padrão é aplicável à maioria desses pacientes, mas as evidências não podem ser consideradas conclusivas, uma vez que pesquisa adicional de alta qualidade provavelmente tenha um impacto modificador na estimativa de efeito.

## INTRODUCTION

Noninvasive ventilation (NIV) was first used in adults by the end of the 1980s.^(1)^ In 1993, a search in the PubMed database using the search term "noninvasive ventilation" would have located only 14 articles. By 2003, the number of studies retrieved with this search term was 88. A search conducted in 2013 resulted in the identification of 230 scientific publications by the same term.^(2)^

Acute severe asthma, also known as status asthmaticus, is essentially a fast and severe exacerbation of asthma that might not respond to standard treatment (oxygen, bronchodilators and steroids).^(3)^ It is characterized by diffuse lower airway obstruction caused by inflammation/edema, in addition to bronchial smooth muscle spasm and mucus plugging, being a reversible condition.^(4)^ Patients exhibit airflow limitation and premature airway closing, which increase the work of breathing. The expiratory phase of breathing becomes active in an attempt to empty the lungs. Increased airway resistance and hyperinflation cause overdistension of the lung parenchyma and chest wall, rendering the next inspiration more difficult.^(4)^ Hyperinflation is dynamic and results in progressively longer time constants, with consequent increases in air trapping and intrinsic positive end-expiratory pressure (PEEP).^(4)^ Severe asthma attacks are acute or subacute episodes of cough, "shortness of breath", "wheezing", "tight chest" or any combination of these symptoms.^(3)^

The rate of hospital admissions caused by asthma among children is approximately 5%; episodes of respiratory failure are uncommon in this population, being developed in 8 to 24% of the asthmatic children admitted to pediatric intensive care units.^(5)^

It is currently believed that in some groups of patients, such as those with exacerbation of chronic obstructive pulmonary disease (COPD), NIV reduces the need for intubation, the mortality rate and the cost of treatment, for which reason its use has become increasingly more frequent.^(6)^

As NIV seems to be efficacious and safe in COPD and the pathophysiology of acute respiratory dysfunction in asthma is similar in many aspects to that of COPD, the use of NIV has been investigated in cases of severe asthma attacks.^(3)^ Nevertheless, the indications for NIV in acute asthma attacks still do not have solid support, and its use has been put into question,^(3)^ particularly in the case of children.

The mechanism of action of NIV in status asthmaticus seems to be based on its bronchodilator effect, which induces alveolar recruitment.^(7)^ The bronchodilator effect is resulted by the use of PEEP, which compensates the effects elicited by the elevation of the intrinsic PEEP. The airflow increases through collateral ventilation channels, resulting in re-expansion of areas with atelectasis and improvement of the ventilation/perfusion ratio, with a consequent reduction in the work of breathing.^(7)^ When applied in bilevel positive airway pressure (BIPAP) mode, the inspiratory positive airway pressure (IPAP) might help the inspiratory muscles to overcome the limitation to the airflow and chest overdistension, thus increasing the tidal volume.^(4)^

In NIV, the patient-machine interface consists of a mask, held in place with a headgear,^(8)^ or nasal prongs, which means that it is without tracheal intubation, reducing the complications associated with invasive mechanical ventilation and becoming an option for patients who are poorly responsive to the standard treatments for status asthmaticus. However, attention should be paid to the general contraindications of NIV, such as altered state of consciousness, hemodynamic instability, gastrointestinal disorders (likely to cause nausea and vomiting), facial trauma, acute failure of more than two organs, among others.^(9)^

This ventilation support is usually provided by continuous (CPAP) or bilevel (BIPAP) positive airway pressure.^(10)^

The aim of the present study was to assess the quality of the available evidence to establish guidelines for use of NIV in the management of status asthmaticus in children unresponsive to standard treatment.

## METHODS

Search, selection and analysis were conducted for all original articles on asthma and NIV in children (up to 18 years old) published until September 1, 2014 in any language in the electronic databases PubMed, Web of Science, Cochrane Library, Scopus and SciELO; the articles were located using the search terms (listed in Health Science Descriptors - Descritores de Ciências da Saúde - DeCs) "asthma", "status asthmaticus", "noninvasive ventilation", "Bronchospasm", "continuous positive airway pressure", "child", "infant", "pediatrics", "hypercapnia", "respiratory failure" and keywords "BIPAP", "CPAP", "Bilevel", "acute asthma" and "near fatal asthma".

The articles located by the search were initially selected based on the information provided in their titles and abstracts. Studies with samples containing individuals with lung disorders other than asthma were excluded. Then, the full texts of the selected articles were analyzed, and the references cited in them were surveyed in search for additional studies that could possibly meet the inclusion criteria and had not been located in the first search. As only a small number of articles met the inclusion criteria, all of them were included in the systematic review through assessment of its methodology.

The methodological quality of the articles was assessed by means of the Grading of Recommendations Assessment, Development and Evaluation (GRADE) system for evaluation of scientific evidence. GRADE was chosen because it is a clear and explicit system that considers the design and execution of studies and their consistency and linear direction in the judgment of the quality of the evidence corresponding to each outcome/relevant consequence.^(11-15)^

In GRADE, the quality of evidence is classified as high, moderate, low or very low (Table 1). Some organizations prefer to analyze the categories low and very low together.

**Table 1:** GRADE quality of evidence^(11-16)^

High (A)	Consistent, with evidence in randomized controlled trials or meta-analyses, without considerable limitations or with exceptionally strong evidence from observational studies. Further research is very unlikely to change the confidence in the estimate of effect.
Moderate (B)	Evidence from randomized controlled trials with considerable limitations (inconsistent results, methodological flaws, imprecision, indirect results). Further research is likely to have an impact on the confidence in the estimate of the effect and might change the estimate.
Low (C)	Evidence of at least one important result in observational studies, case series or randomized controlled trials with serious flaws or indirect evidence. Further research is very likely to have an impact on the confidence in the estimate of the effect and is likely to change the estimate.
Very low (C)	Any estimate of the effect is very uncertain.

Judicious evaluation of the quality of the evidence was independently performed by two reviewers.

## RESULTS

After the database search, only nine articles were located and included in the systemic review (Table 2). These articles were found in duplicate in the investigated databases: two in the Cochrane Library, six in the Web of Science, five in Scopus and eight in PubMed, but none in SciELO. Two articles (22%) had level of evidence A, one (11%) had level of evidence B and six (67%) had level of evidence C (Table 3).

**Table 2:** Main characteristics of the studies

Author	Design	Sample	Intervention	Outcomes	Conclusion
Carroll and Schramm^(10)^	Retrospective, case series (review of medical records)	5 children 2 to 18 years old with SA received NIV as a part of the treatment performed in a PICU from October 2002 to April 2004. Children with conditions other than asthma were not included.	No intervention. Evaluation before onset of NIV and 30 and 60 minutes afterwards (RR, MPIS, oxygen saturation:. BIPAP 12 - 16 X 6 - 8. Patients were allowed 15-minute breaks every 2 hours for comfort, eating and drinking. NIV stopped based on staff assessment. Associated conventional treatment.	4 out the 5 children were morbidly obese; significant ≠: ↓ RR (p = 0.03), clinical improvement according to MPIS (p = 0.03) after onset of NIV. Average NIV duration = 33.2 hours. NIV was well tolerated by all of the children; 1 required sedation.	NIV was well tolerated by this group of children with SA and can improve subjective and objective measures of respiratory dysfunction. NIV may be a useful adjunct in the treatment of SA in children.
Mayordomo-Colunga et al.^(4)^	Prospective observational	72 children over six months old with SA unresponsive to standard treatment, m-WCAS > 4 and ↑ work of breathing; PICU, July 2004 to December 2009. Children with contraindications for NIV were excluded. OTI criterion: no improvement with BIPAP settings up to 20 X 10	Patients evaluated 1, 6, 12, 24 and 48 hours after onset of treatment. Nasal or face mask; short periods without NIV for comfort and aspiration; initial parameters: EPAP 5 and IPAP 6 - 8cmH_2_O; sedation for adaptation as per need; nebulization coupled to the circuit + conventional treatment	Significant p < 0.05; 72 children received NIV; Improvement of m-WCAS, ↓ HR and RR in the first hour (p < 0.01); PaCO_2_ measured before NIV in 13 cases, all showed ↓ PaCO_2_ in the first hour; average NIV duration 33 hours; average length of stay 3 days; face mask in 91.5% of cases; skin lesion (11), gastric distension (4), UA bleeding, barotrauma and subcutaneous emphysema (1 each); sedation in 58.3% (younger children - p < 0.01); 5 OTIs; 1 death after OTI (arrhythmia).	The results show that NIV is a feasible therapy in children with SA unresponsive to conventional treatment.
Akingbola et al.^(22)^	Case report	3 children - 9, 11 and 15 years old, hypercapnic respiratory failure by SA, PICU from 2 institutions	No intervention. Description of cases.	48 hours in ICU; BIPAP 12 to 17 hours; IPAP 10 to 14 and EPAP 4 to 5; BIPAP → ABGs improvement (↑ pH and ↓PaCO_2_), gradual ↓RR	In 3 children with SA, BIPAP seemed to improve ventilation and gas exchange, culminating in resolution of hypercapnic respiratory failure. RCTs assessing NIV in this clinical condition are needed.
Beers et al.^(21)^	Retrospective descriptive	83 children - 2 to 17 years old with acute asthma refractory to conventional treatment treated with BIPAP; pediatric emergency department, from April 1 2003 to August 31 2004.	No intervention. Review of medical records by the principal investigator. BIPAP by nasal mask; children with any comorbidity were excluded from the study.	Average age 8 years old; 64% males; average BIPAP duration: 5.8 hours; 73 tolerated BIPAP - 4 did not tolerate BIPAP from the first seconds - 10 minutes; 77% ↓ RR, 23% with no change; 88% ↑ oxygen saturation, 12% with no change (adequate saturation before BIPAP); 78 admitted to PICU, 2 OTIs. 22% in ward service; no deaths, pneumothorax, pneumomediastinum or epistaxis	The results suggest that addition of BIPAP in treating pediatric SA is safe and well tolerated. This intervention shows promise as a beneficial adjunct to conventional medical treatments. However, future prospective investigation is warranted to confirm these findings.
Needleman et al.^(19)^	Prospective trial	18 patients 8 - 21 years old - SA refractory to conventional treatment in PICU. Did not include children < 8 years old because they tend not to tolerate the mask or children with cardiovascular diseases, pneumothorax or UA obstruction.	BIPAP through nasal mask, initial parameters 10 X 4, ↑ as per tolerance up to 15 X 6 after 10 minutes. RIP before, during and after NIV (10 minutes at each stage: to assess respiratory mechanics.	15 children completed the study (2 excluded for data system malfunction, 1 for discomfort under NIV even with improvement on RIP). After onset of NIV ↓ RR (p = 0.002), fractional inspired time (Ti/Ttot - p = 0.01) and rib cage-abdominal phase angle (p = 0.02) in 12 out of the 15 patients. 3 patients did not exhibit clear response to NIV	NIV is a safe and effective treatment of SA in pediatric patients.
Basnet et al.^(17)^	RCT, prospective, non-blinded	20 patients; PICU; 1 - 18 years old - SA, from January 2009 to January 2010; CAS 3 to 8 after initial pharmacological treatment. Excluded patients without UA protective reflexes or respiratory drive, lesions in or procedures involving the face. No demographic differences between the groups at baseline.	10 patients randomized to receive NIV + standard treatment (GNIV) and 10 patients standard treatment only (Gstandard); BIPAP through nasal or full-face mask, 8 X 5 for VT 6 to 9 mL/ kg; bronchodilator through the circuit as needed; evaluation at 2, 4 - 8, 12 - 16 and 24 hours after onset of intervention by respiratory therapist (not involved in the study) → CAS, RR, HR, need for oxygen and associated treatments.	GNIV: greater improvement on CAS at all time-points of evaluation (p < 0.1), greater ↓ RR and oxygen requirement after 2 hours (p = 0.1 and p = 0.3); children had less need for adjunct therapies (statistically non-significant) and ↓ HR (at 12-16 hours, statistically significant); 9 out the 10 children tolerated NIV (in 1 NIV was discontinued due to persistent cough); all the children attained VT 6 to 9mL/kg with 8 X 5 except for 1 - required full-face mask. Length of stay at PICU similar in both groups.	Early initiation of NIV along with short acting bronchodilators and systemic steroids can be safe, well tolerated and effective in the management of children with SA.
Williams et al.^(20)^	Descriptive, prospective and retrospective	165 children < 20kg; moderate/severe asthma exacerbations; emergency department; age: 0.6 to 8.24 years old. Children who received BIPAP < 30 minutes, born with gestational age < 28 weeks or with chronic disorders were excluded.	Retrospective and prospective description of 112 and 53 patients, respectively.	No deaths, pneumothorax or aspiration pneumonia (1 case with vomiting); 4 OTI even after NIV; 6 patients excluded due to NIV < 30 minutes (agitation; children aged 1 to 4 years old). Improvement on PAS in all of the cases; 99 children were admitted to ICU, 57 to hospital wards and 9 were discharged home.	BIPAP utilization in acute pediatric asthma exacerbations for patients < 20kg is safe and may improve clinical outcomes. These findings warrant future prospective investigation of this subject.
Thill et al.^(18)^	Prospective, crossover randomized	20 children aged 2 months to 14 years old, admitted to PICU along 6 months, with lower airway obstruction and CAS > 3 - < 8; children with tracheostomy, absent airway protection reflexes, abnormalities in or procedures involving the face were excluded; no significant differences between the groups at baseline; no patient with severe hypercapnia.	Patients randomized to receive either 2 hours of NIV followed by crossover to 2 hours of standard treatment alone (G1) or vice-versa (G2). BIPAP through nasal mask, spontaneous mode, backup RR 10, initial parameters 10 X 5, bronchodilator through circuit as per need. Patients independently assessed by principal investigator and respiratory therapist 2 and 4 hours after onset of treatment by means of CAS.	4 patients did not complete the study: 3 OTIs (1 from G1 and 2 from G2); NIV discontinued in 1 patient due to discomfort; no deaths or adverse events; no significant difference between the principal investigator's and respiratory therapist's assessments; ↓ RR, CAS while under NIV in both groups; NIV did not change HR in either group; no significant difference in oxygen saturation or transcutaneous CO_2_; required FiO_2_ + ↓ in the patients under NIV; no child was given sedatives or anxiolytics.	NIV can be an effective treatment for children with acute lower airway obstruction.
Haggenmacher et al.^(23)^	Case report	One 11-month-old infant with SA, respiratory dysfunction, ABGs: pH 7.29 and PaCO_2_ 69; already given oxygen and conventional treatment.	Non-invasive BIPAP through an adult nasal mask used as a pediatric face mask; 11 X 6 initially, IPAP ↑ to 13 after RR 30.	Improvement of respiratory dysfunction and ABGs return to normal values in 72 hours.	NIV + standard treatment is useful for small children with SA refractory to conventional treatment; RCTs are warranted to confirm findings.

SA - status asthmaticus; NIV - noninvasive ventilation; PICU - pediatric intensive care unit; RR - respiratory rate; MPIS - Modified Pulmonary Index Score; BIPAP - bilevel positive airway pressure; m-WCAS - Modified Wood's Clinical Asthma Score; OTI - orotracheal intubation; EPAP - expiratory positive airway pressure; IPAP - inspiratory positive airway pressure; HR - heart rate; PaCO_2_ - partial pressure of arterial carbon dioxide; RCT - randomized clinical trial; UA - upper airways; RIP - Respiratory Inductive Plethysmography; Ti/Ttot - inspired time/total time; CAS -clinical asthma score; GNIV - group noninvasive ventilation; Gstandard - standard group VT - tidal volume; PAS - pediatric asthma score; CO_2_ - carbon dioxide; FiO_2_: fraction of inspired oxygen; ABGs: arterial blood gases

**Table 3:** GRADE system for quality of evidence

Author	High	Moderate	Low	Very low
Basnet et al.^(17)^	X			
Thill et al.^(18)^	X			
Needleman et al.^(19)^		X		
Williams, et al.^(20)^			X	
Beers et al.^(21)^			X	
Mayordomo-Colunga et al.^(17)^			X	
Carroll et al.^(4)^				X
Akingbola et al.^(22)^				X
Haggenmacher et al.^(23)^				X

## DISCUSSION

Few published studies discuss the use of NIV for the management of severe acute asthma in children, and most of them are observational. The only two randomized clinical trials^(17,18)^ have several limitations, such as a lack of blinding participants and investigators and small sample sizes. In 2010, the journal Pediatric Critical Care Medicine published the abstract of a paper presented at a meeting on this subject, which likely corresponds to the early stages of a study published in 2012.^(17)^ This prospective open-label randomized clinical trial compared NIV combined with standard treatment and standard treatment alone for the management of severe acute asthma in children aged one to 18 years old.

One of the randomized clinical trials^(18)^ located in the present review did not exclude the possibility of having included participants with other lower airway obstructive diseases as a function of difficulties in differential diagnosis. Nevertheless, it focused on asthmatic patients and even used a scale for asthma severity assessment (Clinical Asthma Score).

The one prospective study^(19)^ included in the present systematic review used plethysmography as an objective measure for assessment of respiratory mechanics. The authors of that study concluded that NIV is safe and effective for the management of severe acute asthma in the pediatric population.

The remainder of the located studies are observational, consisting of cohorts^(4,20,21)^ and case series/reports.^(10,22,23)^

One of the studies tested the hypothesis that use of a CPAP induces autonomic modulations that increase parasympathetic activation, in addition to bronchodilation resulting from the mechanical effect of positive pressure. The CPAP level used was 10cmH_2_O over 20 minutes. This study^(24)^ found an increase of the vagal tone during CPAP use, with the effect remaining after discontinuation because of activation of the non-cholinergic parasympathetic pathway, with consequent inhibition of bronchoconstriction caused by stimulation of the cholinergic pathway.

The possibility that NIV improves aerosol (bronchodilator) deposition in the airways by inhalation therapy during asthma attacks and exacerbations of COPD was also considered.^(25)^ The results seemingly depend on countless associated factors, such as the type of ventilator, ventilation mode, type of patient-machine interface and position of the aerosol therapy connection in the circuit, among others. Nevertheless, it is believed that combinations of NIV and inhaled medications have beneficial effects, provided proper attention is paid to the application of this technique.

NIV might be associated with some complications, such as skin lesions (from the mask pressure), gastric distention that might cause vomiting and aspiration^(26)^ and subcutaneous emphysema, among others. In clinical practice, such deleterious effects might be minimized through the application of hydrocolloid sheets between the skin and the mask, the use of a nasogastric tube attached to a collector bag, short pauses for face comfort and adjustment of the NIV pressure settings as needed.

Another fact that should be taken into account is that some patients might feel discomfort caused by the air pressure and flow.^(26)^ Some cases require some modality of sedation, which should be thoroughly assessed, as it might cause respiratory depression.

The limitations exhibited by the analyzed studies might derive from the fact that the use of a mask (or other types of interface used in NIV) will certainly be perceived by patients and investigators. In addition, it is very difficult to deliver oxygen alone (with no pressure for a control group) through any kind of NIV interface; mainly because it might cause discomfort and thus likely fails to generate the desired FiO_2_ compared to when normal oxygen therapy systems are used.

There are also ethical issues to consider. Severe acute asthma attacks might be fatal; thus, treatment must be carefully chosen to resolve the attack as soon as possible, which might make simple randomization to receive or to not receive NIV difficult.

The authors of all analyzed studies rated NIV as a safe and efficacious adjuvant treatment for children with status asthmaticus who are unresponsive to conventional treatment; nevertheless, one should take the aforementioned considerations about the methodological quality of the studies into account.

## CONCLUSION

The results suggest that noninvasive ventilation is applicable for the treatment of status asthmaticus in most pediatric patients unresponsive to standard treatment. However, the available evidence cannot be considered as conclusive, as further high-quality research is likely to have impacts on and change the estimates of effects.

## References

[1] Marohn K, Panisello JM (2013). Noninvasive ventilation in pediatric intensive
care. Curr Opin Pediatr.

[2] Argent AC, Biban P (2014). What's new on NIV in the PICU: does everyone in respiratory
failure require endotracheal intubation?. Intensive Care Med.

[3] Papiris S, Kotanidou A, Malagari K, Roussos C (2002). Clinical review: severe asthma. Crit Care.

[4] Mayordomo-Colunga J, Medina A, Rey C, Concha A, Menéndez S, Arcos ML (2011). Non-invasive ventilation in pediatric status asthmaticus: a
prospective observational study. Pediatr Pulmonol.

[5] (2012). Diretrizes da Sociedade Brasileira de Pneumologia e Tisiologia
para o Manejo da Asma - 2012. J Bras de Pneumol.

[6] Schettino GP, Reis MA, Galas F, Park M, Franca S, Okamoto V (2007). Mechanical ventilation noninvasive with positive pressure. J Bras Pneumol.

[7] Carson KV, Usmani ZA, Smith BJ (2014). Noninvasive ventilation in acute severe asthma: current evidence
and future perspectives. Curr Opin Pulm Med.

[8] Pons Odena M, Cambra Lasaosa FJ, Sociedad Española de Cuidados Intensivos (2003). PediátricosMechanical ventilation in pediatrics (III).
Weaning, complications and other types of ventilation. Noninvasive ventilation]. An Pediatr (Barc).

[9] James MM, Beilman GJ (2012). Mechanical ventilation. Surg Clin North Am.

[10] Carroll CL, Schramm CM (2006). Noninvasive positive pressure ventilation for the treatment of
status asthmaticus in children. Ann Allergy Asthma Immunol.

[11] Guyatt GH, Oxman AD, Kunz R, Falck-Ytter Y, Vist GE, Liberati A, Schünemann HJ, GRADE Working Group (2008). Going from evidence to recommendations. BMJ.

[12] Guyatt GH, Oxman AD, Kunz R, Jaeschke R, Helfand M, Liberati A, Vist GE, Schünemann HJ, GRADE Working Group (2008). Incorporating considerations of resources use into grading
recommendations. BMJ.

[13] Guyatt GH, Oxman AD, Vist GE, Kunz R, Falck-Ytter Y, Alonso-Coello P, Schünemann HJ, GRADE Working Group (2008). GRADE Wan emerging consensus on rating quality of evidence and
strength of recommendations. BMJ.

[14] Atkins D, Best D, Briss PA, Eccles M, Falck-Ytter Y, Flottorp S, Guyatt GH, Harbour RT, Haugh MC, Henry D, Hill S, Jaeschke R, Leng G, Liberati A, Magrini N, Mason J, Middleton P, Mrukowicz J, O'Connell D, Oxman AD, Phillips B, Schünemann HJ, Edejer T, Varonen H, Vist GE, Williams JW, Zaza S, GRADE Working Group (2004). Grading quality of evidence and strength of
recommendations. BMJ.

[15] Guyatt GH, Oxman AD, Kunz R, Vist GE, Falck-Ytter Y, Schünemann HJ, GRADE Working Group (2008). What is "quality of evidence" and why is it important to
clinicians. BMJ.

[16] Guyatt GH, Norris SL, Schulman S, Hirsh J, Eckman MH, Akl EA, Crowther M, Vandvik PO, Eikelboom JW, McDonagh MS, Lewis SZ, Gutterman DD, Cook DJ, Schünemann HJ, American College of Chest Physicians (2012). Methodology for the development of antithrombotic therapy and
prevention of thrombosis guidelines: Antithrombotic Therapy and Prevention
of Thrombosis, 9th ed: American College of Chest Physicians Evidence-Based
Clinical Practice Guidelines. Chest.

[17] Basnet S, Mander G, Andoh J, Klaska H, Verhulst S, Koirala J (2012). Safety, efficacy, and tolerability of early initiation of
noninvasive positive pressure ventilation in pediatric patients admitted
with status asthmaticus: a pilot study. Pediatr Crit Care Med.

[18] Thill PJ, McGuire JK, Baden HP, Green TP, Checchia PA (2004). Noninvasive positive-pressure ventilation in children with lower
airway obstruction. Pediatr Crit Care Med.

[19] Needleman JP, Sykes JA, Schroeder SA, Singer LP (2004). Noninvasive positive pressure ventilation in the treatment of
pediatric status asthmaticus. Pediatr Asthma Allergy Immunol.

[20] Williams AM, Abramo TJ, Shah MV, Miller RA, Burney-Jones C, Rooks S (2011). Safety and clinical findings of BiPAP utilization in children 20
kg or less for asthma exacerbations. Intensive Care Med.

[21] Beers SL, Abramo TJ, Bracken A, Wiebe RA (2007). Bilevel positive airway pressure in the treatment of status
asthmaticus in pediatrics. Am J Emerg Med.

[22] Akingbola OA, Simakajornboon N, Hadley EF, Hopkins RL (2002). Noninvasive positive-pressure ventilation in pediatric status
asthmaticus. Pediatr Crit Care Med.

[23] Haggenmacher C, Biarent D, Otte F, Fonteyne C, Clement S, Deckers S (2005). Non-invasive bi-level ventilation in paediatric status
asthmaticus. Arch Pediatr.

[24] de Freitas Dantas Gomes EL, Costa D, Germano SM, Borges PV, Sampaio LM (2013). Effects of CPAP on clinical variables and autonomic modulation in
children during an asthma attack. Respir Physiol Neurobiol.

[25] Dhand R (2012). Aerosol therapy in patients receiving noninvasive positive
pressure ventilation. J Aerosol Med Pulm Drug Deliv.

[26] Gay PC (2009). Complications of noninvasive ventilation in acute
care. Respir Care.

